# Global Reliability Sensitivity Analysis Based on Maximum Entropy and 2-Layer Polynomial Chaos Expansion

**DOI:** 10.3390/e20030202

**Published:** 2018-03-16

**Authors:** Jianyu Zhao, Shengkui Zeng, Jianbin Guo, Shaohua Du

**Affiliations:** 1 School of Reliability and Systems Engineering, Beihang University, Beijing 100191, China; 2 Science and Technology on Reliability and Environmental Engineering Laboratory, Beihang University, Beijing 100191, China; 3 CRRC ZIC Research Institute of Electrical Technology & Material Engineering, Zhuzhou 412001, China

**Keywords:** global reliability sensitivity analysis, polynomial chaos expansion, Sobol’s indices, the maximum entropy method

## Abstract

To optimize contributions of uncertain input variables on the statistical parameter of given model, e.g., reliability, global reliability sensitivity analysis (GRSA) provides an appropriate tool to quantify the effects. However, it may be difficult to calculate global reliability sensitivity indices compared with the traditional global sensitivity indices of model output, because statistical parameters are more difficult to obtain, Monte Carlo simulation (MCS)-related methods seem to be the only ways for GRSA but they are usually computationally demanding. This paper presents a new non-MCS calculation to evaluate global reliability sensitivity indices. This method proposes: (i) a 2-layer polynomial chaos expansion (PCE) framework to solve the global reliability sensitivity indices; and (ii) an efficient method to build a surrogate model of the statistical parameter using the maximum entropy (ME) method with the moments provided by PCE. This method has a dramatically reduced computational cost compared with traditional approaches. Two examples are introduced to demonstrate the efficiency and accuracy of the proposed method. It also suggests that the important ranking of model output and associated failure probability may be different, which could help improve the understanding of the given model in further optimization design.

## 1. Introduction

Sensitivity analysis (SA) is one of the most important methodologies dealing with optimization in engineering practice. It aims to quantify importance in the output of a model with regards to some input parameters or design variables [1,2,3,4]. With the results of SA, important variables of given model are chosen for further study when less important variables may be treated as constant. This could help reduce the dimension of inputs (number of input variables) and improve the model understanding.

Generally, SA methodologies can be classified into local sensitivity analysis (LSA) and global sensitivity analysis (GSA) [1,5]. The former only concentrate on the influence of single input parameter on model output by gradient estimating in a small neighborhood around a normal point. It is often used to provide deterministic direction to be close to the optimal object [6]. Meanwhile, the latter one tries to quantify the output uncertainty when all input parameters are varying over entire value domain. And it does not depend on the choice of a nominal point [5,7]. Thus, GSA is suitable for uncertainty quantification (UQ) and reliability based design optimization (RBDO) [8].

Numerous methodologies are developed for GSA, among which variance-based methods are widely used [1,5]. Variance-based methods take the well-known variance decomposition formula *V*(*Y*) = *V*(*E*(*Y*|*X*)) + *E*(*V*(*Y*|*X*) as their foundation. This formula has variance separated by using the variance of conditional expectation and associated residual variance. Thus, the contribution or importance of *X* on *Y* could be further studied. Many researchers take Sobol’s indices because they could provide a clear, accurate and robust indication of the selected uncertain inputs both quantitatively and qualitatively [9]. Although Sobol’s indices are traditionally applied for non-statistical model output, some researchers have extended Sobol’s indices to identify uncertainty effects of inputs on reliability. Morio only studied the effects of some variable distribution parameters, such as variable means, but it did not consider the variable uncertainties themselves [10]. Borgonovo proposed the moment-independent global sensitivity indices which are derived from conditional probability density function (PDF) [11,12]. Lu accepted this idea and further developed the variance-based importance measurement for global reliability sensitivity analysis (GRSA) with associated global reliability sensitivity indices [13,14]. Cui and Li proved the equivalence relation between these global reliability sensitivity indices and Sobol’s indices when taking the indicator function as the assumed model output. 

Unlike traditional global sensitivity indices calculation, the global reliability sensitivity indices aim at calculating importance of input variables on reliability that is a statistical parameter. These indices are specifically concerned about the variance of conditional expectation of indicator function according to variance decomposition formula. However, the indicator function maps every single model output to only two real numbers 0 or 1 discretely, so the analytical method and surrogate method, which are popular with uncertainty analysis, have not been developed for GRSA because it is difficult to obtain an analytical formula of the indicator function. In this way, the Crude Monte Carlo Simulation (MCS) is always employed to calculate such measurement [15,16], which leads that the computation cost is unacceptable when the given model is complex. Although Li et al. used the importance sampling technologies to improve sampling efficiency [13], quite a few samples are inevitable.

Fortunately, the conditional expectation of indicator function is equal to the conditional failure probability (CFP) that could be regarded as a continuous random variable. Thus, the variance of conditional expectation of indicator function could be replaced by the variance of CFP. In this way, if CFP could be assumed as the model output, there might be an unknown expression between CFP and corresponding conditional variable. So, the surrogate method could be used to approximate the CFP. Then, the statistical properties of CFP, such as mean and variance, may be calculated by surrogate model efficiently.

As for the issue of the statistical properties of a surrogate model, polynomial chaos expansions (PCE) technology provides a powerful tool for this application [17,18]. The PCE is a surrogate modelling technology with orthogonal polynomial basis functions, and it has statistical moments calculation simplified. In other words, variance information of surrogate model could be readily obtained by post-processing PCE’s coefficients analytically [19,20]. Therefore, it is suitable to make a surrogate model for CFP and calculate its variance. However, a challenge for PCE method in such application is that quite a few specific probability values are required. In addition, since the CFP is dependent on a set of conditional variables, each global reliability sensitivity index corresponding to different conditional variables requires a specific surrogate model. This may be time consuming when computing several indices. To circumvent this problem, the maximal entropy (ME) method is introduced since it could compute probabilities precisely and analytically only with prior moment information of model output [21,22,23,24]. Considering the orthogonal characteristics, the PCE could not only provide the first two order moments directly, but also provide higher order moments by PCE multiplication algorithm without additional sampling. In this way, the combination of PCE and ME could make GRSA efficiently. To the author’s knowledge, this idea has not been developed for global reliability sensitivity indices calculation.

Therefore, a new 2-layer PCE algorithm coupling PCE and ME is developed. That is, the first layer PCE is to generate a surrogate model by traditional algorithm. The second layer PCE is to construct several surrogate models for CFPs with the help of ME, which are referred as reliability polynomial chaos expansions (R-PCEs). The moment information that ME requires is provided by the first layer PCE when the conditional variables are given and fixed. Then, the variance of each CFP comes out by analytically post processing R-PCE coefficients, followed by global reliability sensitivity indices.

The presentation of the work is structured as follows: Section 2 provides a brief introduction of GSA and GRSA respectively. Section 3 presents the proposed 2-layer PCE framework and detailed calculation procedure. A numerical example and an engineering example are presented to test the performance of the proposed method in Section 4, while Section 5 summarizes the main contributions of this paper. The symbols used in this paper are listed in Table A1 in Appendix A.

## 2. Related Work

### 2.1. Global Sensitivity Analysis and Sobol’s Indices

In this section, the traditional variance based GSA is briefly reviewed [16,19]. Let us consider a model *y* = *g*(***x***) with *n*-dimensional independent identical distribution random input ***x*** = (*x*_1_, …, *x_n_*) ∈*Ω^n^* and a scalar output *y*. Denote *f_x_*(***x***) as the joint probability density function (PDF), then we have $f_{x}\left(x\right)=\prod_{i=1}^{n}f_{x}\left(x_{i}\right)$. In addition, we assume that $x\in \Omega^{n}\mapsto g\left(x\right)\in L^{2}\left(\Omega^{n},f_{x}\left(x\right)\right)$.

The Sobol’s decomposition of *g*(***x***) can be expressed as:

(1) $$g\left(x\right)=g_{0}+\sum_{i=1}^{n}g_{i}\left(x_{i}\right)+\sum_{1\leq i<j\leq n}g_{ij}\left(x_{i},x_{j}\right)+\cdots +g_{1,2,\cdots ,n}\left(x_{1},\cdots ,x_{n}\right),$$

where:

(2) $$\begin{array}{l} g_{0}=\int_{\Omega^{n}}g\left(x\right)f_{x}\left(x\right)dx,\\ g_{i}\left(x_{i}\right)=\int_{\Omega^{n-1}}g\left(x\right)f_{x}\left(x_{\sim [i]}\right)dx_{\sim [i]}-g_{0},\\ g_{ij}\left(x_{i},x_{j}\right)=\int_{\Omega^{n-2}}g\left(x\right)f_{x}\left(x_{\sim [i,j]}\right)dx_{\sim [i,j]}-g_{i}\left(x_{i}\right)-g_{j}\left(x_{j}\right)-g_{0}, \end{array}$$

and so on ***x***_~[*i*]_ in Equation (2) means the vector without the element of *i*, that is, ***x***_~[*i*]_ = (*x*_1_, …, *x*_*i*−1_, *x*_*i*+1_, …, *x_n_*).

Furthermore, the expansion items above are orthogonal to each other, that is:

(3) $$\int_{\Omega^{n}}g_{1,2,\cdots ,i_{s}}\left(x_{1},x_{2},\cdots ,x_{i_{s}}\right)g_{1,2,\cdots ,j_{t}}\left(x_{1},x_{2},\cdots ,x_{j_{t}}\right)f_{x}\left(x\right)dx=0\;\forall \;\left(x_{1},x_{2},\cdots ,x_{i_{s}}\right)\neq \left(x_{1},x_{2},\cdots ,x_{j_{t}}\right).$$

And the variance of output *y* = *g*(***x***) is defined as:

(4) $$V\left(g\left(x\right)\right)=\int_{\Omega^{n}}{\left(g\left(x\right)-g_{0}\right)}^{2}f_{x}\left(x\right)dx.$$

Sobol’s proposed a variance-based representation to measure the importance of input variable. With Equations (1), (3) and (4), the Sobol’s indices are given as:

(5) $$S_{i_{1},\cdots ,i_{s}}=\frac{V\left(g_{i_{1},\cdots ,i_{s}}\left(x_{i_{1}},\cdots ,x_{i_{s}}\right)\right)}{V\left(g\left(x\right)\right)}=\frac{\int_{\Omega^{s}}g_{i_{1},\cdots ,i_{s}}^{2}\left(x_{i_{1}},\cdots ,x_{i_{s}}\right)f_{x}\left(x_{i_{1}},\cdots ,x_{i_{s}}\right)dx_{i_{1}}\cdots x_{i_{s}}}{\int_{\Omega^{n}}{\left(g\left(x\right)-g_{0}\right)}^{2}f_{x}\left(x\right)dx},$$

where $V\left(g_{i_{1},\cdots ,i_{s}}\left(x_{i_{1}},\cdots ,x_{i_{s}}\right)\right)$ is called as the partial variance, which is distinguished from *V*(*g*(***x***)) above.

Generally, *S_i_* is the first-order sensitivity index, *S_ij_* is the second-order sensitivity index, etc. There are 2*^n^* − 1 Sobol’s sensitivity indices, and they are subjected to the following formula:

(6) $$\sum_{i=1}^{n}S_{i}+\sum_{1\leq i<j\leq n}S_{ij}+\cdots S_{1,2,\cdots ,n}=1.$$

Moreover, since *S_i_* represents the single contribution to the output uncertainty only due to an input *x_i_*, it is also called as the main effect. And the total effect index $S_{T_{i}}$ has been defined to measure the total contribution of an input *x_i_*. It could be calculated as follows:

(7) $$S_{T_{i}}=\sum_{L_{i}}S_{i_{1},\cdots ,i_{s}}=1-S_{\sim [i]},\;\forall \;L_{i}=\left\{\left(i_{1},\cdots ,i_{s}\right):\exists k,1\leq k\leq s,i_{k}=i\right\},$$

where *S*_~[*i*]_ is the sum of all $S_{i_{1},\cdots ,i_{s}}$ without subscribe index *i*.

Traditionally, the Sobol’s indices could be solved by MCS [16], which may be computationally demanding. Sudret proposed a PCE-based method to mitigate this problem with much less computational costs [18].

PCE, which was originally introduced by Wiener [25], employs the Hermite polynomials in the random space to approximate the Gaussian stochastic processes [19,26]. In [20], Xiu and Karniadakis developed the PCE under Wiener-Askey scheme that could be applied to non-Gaussian scenarios. It can uniformly approximate any random process with finite second-order moments. Specifically, the PCE approximation of model *y* = *g*(***x***) is:

(8) $$\begin{array}{ll} y\approx g_{PCE}\left(\xi \right)= & c_{0}+\sum_{i=1}^{n}\sum_{\alpha \in \varphi_{i}}c_{\alpha }\psi_{\alpha }\left(\xi_{i}\right)+\sum_{1\leq i_{1}<i_{2}\leq n}\sum_{\alpha \in \varphi_{i_{1}i_{2}}}c_{\alpha }\psi_{\alpha }\left(\xi_{i_{1}},\xi_{i_{2}}\right)+\cdots +\\ & \sum_{1\leq i_{1}<\cdots <i_{s}\leq n}\sum_{\alpha \in \varphi_{i_{1}\cdots i_{s}}}c_{\alpha }\psi_{\alpha }\left(\xi_{i_{1}},\cdots ,\xi_{i_{s}}\right)+\cdots +\sum_{\alpha \in \varphi_{1,2,\cdots ,n}}c_{\alpha }\psi_{\alpha }\left(\xi_{1},\cdots ,\xi_{n}\right), \end{array}$$

where ***ξ*** *=* (*ξ*_1_, *ξ*_2_, …, *ξ_n_*) is independent standardized orthogonal random variables associated with input ***x*** = (*x*_1_, …, *x_n_*), the subscript ***α*** is a tuple defined as ***α*** *=* (*α_1_*, …, *α_n_*), and $\varphi_{i_{1},\cdots ,i_{s}}$ is defined as a realization of ***α*** tuple in which only the indices {*i*_1_, …, *i_s_*} are nonzero:

(9) $$\varphi_{i_{1},\cdots ,i_{s}}=\left\{\alpha :\;\begin{array}{c} \alpha_{k}>0\;\forall k=1,2,\cdots ,n.\;k\in \left(i_{1},\cdots ,i_{s}\right)\\ \alpha_{k}=0\;\forall k=1,2,\cdots ,n.\;k\notin \left(i_{1},\cdots ,i_{s}\right) \end{array}\right\}.$$

Moreover, $\phi_{k}$ is the *k*th one-dimensional orthogonal polynomials from the Askey scheme. $\psi_{\alpha }\left(\xi_{i_{1}},\cdots ,\xi_{i_{s}}\right)$ is the multi-dimensional PCE basis, which is constructed by tensor products of the corresponding one-dimensional polynomials, namely, $\psi_{\alpha }\left(\xi_{i_{1}},\cdots ,\xi_{i_{s}}\right)=\prod_{k=1}^{i_{s}}\phi_{\alpha_{k}}\left(\xi_{k}\right)$. Besides, the highest degree of orthogonal polynomials in Equation (8) is a predefined PCE degree *p*. Therefore, we have |***α***| ≤ *p*. It should be noted that *p* could be determined by considering the balance of accuracy and efficiency. Specifically, PCEs with small degrees *p* and *p* + 1 could be obtained respectively, which is followed by comparing the difference (usually the first two order moments). If the difference is smaller than a pre-defined threshold, the PCE with *p* + 1 degree is adopted. Otherwise, the PCE with *p* + 2 degree is used to repeat this procedure.

Generally, Equation (8) can be rewritten as:

(10) $$y\approx g_{PCE}\left(\xi \right)=\sum_{j=0}^{N-1}c_{j}\psi_{j}\left(\xi \right),\;for\;\xi =\left(\xi_{1},\cdots ,\xi_{n}\right),$$

where coefficient *c_j_* and expansion base *ψ_j_*(***ξ***) are corresponding to Equation (8) sequentially, $N=\left(\begin{array}{c} n+p\\ p \end{array}\right)=\frac{\left(n+p\right)!}{n!p!}$ is the number of PCE items. Thus, the main effect index *S_i_* and the total effect index $S_{T_{i}}$ could be solved directly with PCE coefficients as:

(11) $$S_{i}=\frac{\sum_{\alpha \in \varphi_{i}}c_{\alpha }^{2}E\left[\psi_{\alpha }^{2}\right]}{\sum_{j=1}^{N-1}c_{j}^{2}E\left(\psi_{j}^{2}\left(\xi \right)\right)},$$

(12) $$S_{T_{i}}=\sum_{\left(i_{1},\cdots ,i,\cdots ,i_{s}\right)\in \varphi_{i_{1},\cdots ,i_{s}}}S_{i_{1},\cdots ,i,\cdots i_{s}}.$$

For more details see Appendix B.

### 2.2. Global Reliability Sensitivity Analysis

If the given model *g*(***x***) above is the limit-state function, the input ***x*** falls in the failure region if *g*(***x***) returns a negative value. Denote *P_f_* as the failure probability, then we have:

(13) $$\begin{array}{ll} P_{f} & =P\left(g\left(x\right)<0\right)\\ & =\int \cdots \int_{g\left(x\text{<}\right)}f_{x}\left(x_{1},x_{2},\cdots ,x_{n}\right)dx_{1}dx_{2}\cdots dx_{n}\\ & =\int \cdots \int_{\Omega^{n}}I_{F}f_{x}\left(x_{1},x_{2},\cdots ,x_{n}\right)dx_{1}dx_{2}\cdots dx_{n}\\ & =E\left[I_{F}\right], \end{array}$$

where $I_{F}=\{\begin{array}{cc} 1, & g\left(x\right)\leq 0,\\ 0, & g\left(x\right)>0. \end{array}$ is the indicator function of the failure domain, and *E*(*I_F_*) is the mean of indicator function.

Similarly, the CFP is defined as follows:

(14) $$P_{f|x_{I}}=E\left[I_{F}|x_{I}\right],$$

where ***x_I_*** is the vector of conditional input variable with subscript ***I*** = {*i*_1_, …, *i_m_*} (1 ≤ *i*_1_ ≤…≤ *i_m_*≤ *n*), $I_{F}|x_{I}=\{\begin{array}{cc} 1, & g\left(x|x_{I}\right)\leq 0,\\ 0, & g\left(x|x_{I}\right)>0. \end{array}$ is the conditional indicator function of the failure domain of the conditional limit-state function *g*(***x*|*x_I_***) and it can be obtained by assuming ***x_I_*** at a fixed realization value while other inputs are free.

Cui and Lü put forward the moment-based importance measure $\delta_{I}^{P}$ of the basic variable on the distribution as [14]:

(15) $$\delta_{I}^{P}=\int_{-\infty }^{+\infty }{\left(P_{f}-P_{f|x_{I}}\right)}^{2}f_{x}\left(x_{I}\right)dx_{I}=E{\left[P_{f}-P_{f|x_{I}}\right]}^{2},$$

where $\delta_{I}^{P}$ eflects the effect of conditional variable ***x_I_*** on failure probability when ***x_I_*** takes its value according to its PDF. Furthermore, Cui proved that:

(16) $$\delta_{I}^{P}=V\left[E\left(I_{F}|x_{I}\right)\right].$$

In this way, $\delta_{I}^{P}$ is equal to the Sobol’s index when *I_F_* is taken as the model output. Therefore, Cui proposed that the main effect index and the total effect index of *x_i_* can be defined as:

(17) $$S_{i}^{\left(R\right)}=\frac{V\left(E\left(I_{F}|x_{i}\right)\right)}{V\left(I_{F}\right)},$$

(18) $$S_{T_{i}}^{\left(R\right)}=1-\frac{V\left(E\left(I_{F}|x_{\sim \left[i\right]}\right)\right)}{V\left(I_{F}\right)},$$

where *V*(*I_F_*), *V*(*E*(*I_F_*|*x_i_*)) and *V*(*E*(*I_F_*|***x***_~[*i*]_)) are expressed as follows:

(19) $$V\left(I_{F}\right)=E\left(I_{F}^{2}\right)-E{\left(I_{F}\right)}^{2}=E\left(I_{F}\right)-E{\left(I_{F}\right)}^{2}=P_{f}-P_{f}^{2},$$

(20) $$V\left(E\left(I_{F}|x_{i}\right)\right)=E\left(E{\left(I_{F}|x_{i}\right)}^{2}\right)-E{\left(E\left(I_{F}|x_{i}\right)\right)}^{2}=E\left(E{\left(I_{F}|x_{i}\right)}^{2}\right)-P_{f}^{2},$$

(21) $$V\left(E\left(I_{F}|x_{\sim \left[i\right]}\right)\right)=E\left(E{\left(I_{F}|x_{\sim \left[i\right]}\right)}^{2}\right)-E{\left(E\left(I_{F}|x_{\sim \left[i\right]}\right)\right)}^{2}=E\left(E{\left(I_{F}|x_{\sim \left[i\right]}\right)}^{2}\right)-P_{f}^{2}.$$

The MCS method and associated improved Importance Sampling (IS) method for solving $S_{i}^{\left(R\right)}$ and $S_{T_{i}}^{\left(R\right)}$ are presented in [27], where the IS method uses the advanced first-order second-moment method [28] to help construct importance sampling density functions within MCS framework. However, they are both time-consuming due to the sampling principle, and the computational burden becomes worse when given model are complex. In this way, a new algorithm is proposed to mitigate this problem.

## 3. R-PCE Framework for GRSA

### 3.1. PCE-Based GRSA Method

Take the limit-state function in Section 2.2 into consideration. As mentioned above, the model output for global reliability sensitivity indices calculation is *I_F_*, and it is difficult to compute *I_F_* related value except for MCS. This is due to the fact that *I_F_* only maps every single model output to two discrete real numbers 0 or 1. Either analytical methods or surrogate methods are efficient when they are applied to continuous model output rather than the discrete case. However, *E*(*I_F_*|*x_i_*) and *E*(*I_F_*|***x***_~[*i*]_) could be replaced by two CFP values *P_f_*(*x_i_*) and *P_f_*(***x***_~[*i*]_) respectively, which could be treated as continuous random variables. In this way, the key point of global reliability sensitivity index calculation is to compute variance values *V*(*P_f_*(*x_i_*)) and *V*(*P_f_*(***x***_~[*i*]_)). Equations (17) and (18) are replaced by Equations (22) and (23):

(22) $$S_{i}^{\left(R\right)}=\frac{V\left(E\left(I_{F}|x_{i}\right)\right)}{V\left(I_{F}\right)}=\frac{E\left(P_{f}{\left(x_{i}\right)}^{2}\right)-P_{f}^{2}}{P_{f}-P_{f}^{2}},$$

(23) $$S_{T_{i}}^{\left(R\right)}=1-\frac{V\left(E\left(I_{F}|x_{\sim \left[i\right]}\right)\right)}{V\left(I_{F}\right)}=1-\frac{E\left(P_{f}{\left(x_{\sim \left[i\right]}\right)}^{2}\right)-P_{f}^{2}}{P_{f}-P_{f}^{2}}.$$

Since the PCE technology provides an efficient surrogate modeling tool to determinate the mean as well as the variance of the model output, a PCE-based method is developed for this implement. However, several probability values required during surrogate modeling procedure may cause computational demanding. So, the analytical ME method is integrated with PCE to solve this problem. Thus, the surrogate model of CFP could be developed, which is referred as R-PCE in this work.

**Figure 1:**
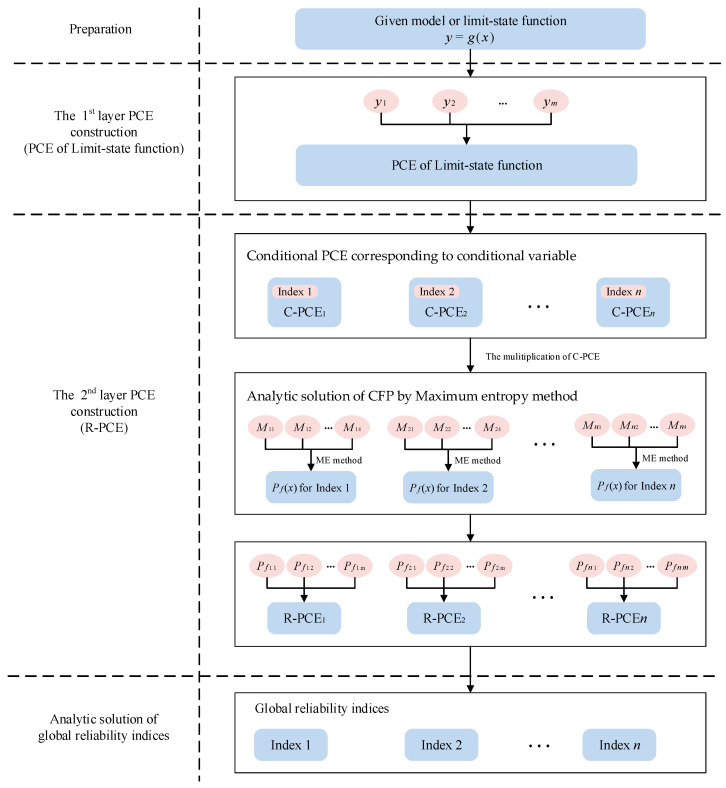
PCE based GRSA method framework.

The PCE-based GRSA method involves a 2-layer PCE surrogate model construction process. The first layer PCE is to generate traditional surrogate model approximating the limit-state function. It is used to obtain mean and variance when given conditional variables are fixed in specific values. And the second layer is to construct R-PCEs for different CFPs. To obtain probability response during the surrogate modeling of R-PCE, the ME method is taken to analytically compute these probability values with the first layer PCE. Besides, since each CFP is dependent on a different conditional variable, each global reliability sensitivity index has its own R-PCE formula. The algorithm framework is shown in Figure 1, and the whole procedure consists of four phases.

The first phase is to prepare inputs or data for GRSA.

In the second phase, the PCE technology is an efficient and accurate tool to approximate the given model with finite samples. Thus, the original model is replaced by the 1st-layer PCE in the following procedures. Besides, the first and second moment of the model output are obtained directly without traditional Mote Carlo sampling.

In the third phase, the PCE technology is developed to make the surrogate model for CFP. Unlike the surrogate model in the second phase, quite a few failure probabilities of given model require to be calculated. Unlike the model output which may be obtained by simulating directly, it is usually difficult to obtain these statistical values. Fortunately, this problem could be overcome by the ME method. According to ME, the probability distribution that best represents the current state of knowledge, which is usually presented in terms of the first *n*-order moments, is the one having the largest entropy [24]. Under this principle, the probability distribution of the model output could be analytically obtained with moment information based on the 1st-layer PCE. The details of this procedure are presented as follows.

First of all, for each global reliability sensitivity index, a set of conditional variable samples is generated by the probabilistic collocation method (PCM). Second, due to the regularity of orthogonal polynomials, the conditional variable is removed from the 1st-layer PCE. Also, the specific formula of the 1st-layer PCE with other variables could remain the form of linear combination of orthogonal polynomials, so does the statistical characteristic. For simplicity, we call it conditional PCE (C-PCE). Thus, the associated moment evaluations are also available. Third, the ME method is taken to solve failure probabilities with regards to specific conditional variable. Fourth, a set of conditional variable samples and associated conditional failure probabilities are prepared for following 2nd-layer PCE (R-PCE) construction, and this procedure is similar to PCE in the second phase. In this way, R-PCE corresponding to specific conditional variable is generated.

Finally, with the coefficients of R-PCE, we could obtain variance of each CFP and calculate global reliability sensitivity index. During the procedure, the number of sampling of both the 1st-layer PCE and the 2nd-layer PCE of proposed method is limited according to PCE sample principle. This makes proposed method efficient. The following section will provide details of how to construct the proposed 2-layer PCE.

### 3.2. Conditional Polynomial Chaos Expansion and Moments Calculation

Without loss of generality, PCE formula of given limit-state function is the same as Equation (8). If the value of *ξ**_I_*** is given as the conditional variable, Equation (8) could be simplified as:

(24) $$\begin{array}{ll} g_{PCE}\left(\xi |\xi_{I}\right)= & {c^{\prime }}_{0}+\sum_{i=1}^{n-\left|I\right|}\sum_{\alpha \in \varphi_{i\notin I}}{c^{\prime }}_{\alpha }\psi_{\alpha }\left(\xi_{i}\right)\\ & +\sum_{1\leq i_{1}<\cdots <i_{s}\leq n-\left|I\right|}\sum_{\alpha \in \varphi_{\left\{i_{1}\cdots i_{s}\right\}\notin I}}{c^{\prime }}_{\alpha }\psi_{\alpha }\left(\xi_{i_{1}},\cdots ,\xi_{i_{s}}\right)+\cdots \\ & +\sum_{\alpha \in \varphi_{\left\{1,2,\cdots ,n-\left|I\right|\right\}\notin I}}{c^{\prime }}_{\alpha }\psi_{\alpha }\left(\xi_{1},\cdots ,\xi_{n-\left|I\right|}\right), \end{array}$$

where |***I***| is the number of elements in subscript ***I***, {*i*_1_, …, *i_s_*}∉***I*** represents that $\forall i_{k}\in \left\{i_{1},\cdots ,i_{s}\right\},i_{k}\notin I$. The coefficients are subject to:

(25) $${c^{\prime }}_{0}=c_{0}+\sum_{\forall I^{\prime }\in I,I^{\prime }\notin \emptyset ,\alpha \in \varphi_{I^{\prime }}}c_{\alpha }\psi_{\alpha }\left(\xi_{I^{\prime }}\right)=c_{0}+\sum_{\forall I^{\prime }=\left\{i_{1},\cdots ,i_{k}\right\}\in I,I^{\prime }\notin \emptyset ,\alpha \in \varphi_{I^{\prime }}}c_{\alpha }\psi_{\alpha }\left(\xi_{i_{1}},\cdots ,\xi_{i_{k}}\right)$$

and:

(26) $${c^{\prime }}_{\alpha }\psi_{\alpha }\left(\xi_{i_{1}},\cdots ,\xi_{i_{s}}\right)=\sum_{\forall I^{\prime }\in I,I^{\prime }\notin \emptyset ,\alpha \in \varphi_{i_{1}\cdots i_{s},I^{\prime }}}c_{\alpha }\psi_{\alpha }\left(\xi_{i_{1}},\cdots ,\xi_{i_{s}},\xi_{I^{\prime }}\right).$$

The total number of coefficients is $N^{\prime }=\left(\begin{array}{c} n+p-\left|I\right|\\ p \end{array}\right)=\frac{\left(n+p-\left|I\right|\right)!}{\left(n-\left|I\right|\right)!p!}$. Equation (24) is called as C-PCE. Thus, the mean and variance could be obtained by the following equations:

(27) $$E\left(g_{PCE}\left(\xi |\xi_{I}\right)\right)={c^{\prime }}_{0},$$

(28) $$\begin{array}{ll} V\left(g_{PCE}\left(\xi |\xi_{I}\right)\right)= & \sum_{i=1}^{n-\left|I\right|}\sum_{\alpha \in \varphi_{i\notin I}}{\left({c^{\prime }}_{\alpha }\right)}^{2}E\left(\psi_{\alpha }^{2}\left(\xi_{i}\right)\right)+\cdots \\ & +\sum_{1\leq i_{1}<\cdots <i_{s}\leq n-\left|I\right|}\sum_{\alpha \in \varphi_{\left\{i_{1}\cdots i_{s}\right\}\notin I}}{\left({c^{\prime }}_{\alpha }\right)}^{2}E\left(\psi_{\alpha }^{2}\left(\xi_{i_{1}},\cdots ,\xi_{i_{s}}\right)\right)+\cdots \\ & +\sum_{\alpha \in \varphi_{\left\{1,2,\cdots ,n-\left|I\right|\right\}\notin I}}{\left({c^{\prime }}_{\alpha }\right)}^{2}E\left(\psi_{\alpha }^{2}\left(\xi_{1},\cdots ,\xi_{n-\left|I\right|}\right)\right). \end{array}$$

To simplify the expression, C-PCE could be rewritten as:

(29) $$g_{PCE}\left(\xi |\xi_{I}\right)=\sum_{j=0}^{N^{\prime }-1}{c^{\prime }}_{j}\psi_{\alpha }\left(\xi |\xi_{I}\right),$$

where ${c^{\prime }}_{j}$ and *ψ_α_*(***ξ***|***ξ_I_***) are corresponding to Equation (28) sequentially, notation “***ξ***|***ξ_I_***” in *ψ_α_*(***ξ***|***ξ_I_***) means that expansion base does not include conditional variable ***ξ_I_***.

The mean and variance of C-PCE are given as Equations (30) and (31) respectively:

(30) $$E\left(g_{PCE}\left(\xi |\xi_{I}\right)\right)={c^{\prime }}_{0},$$

(31) $$V\left(g_{PCE}\left(\xi |\xi_{I}\right)\right)=\sum_{j=1}^{N^{\prime }-1}{c^{\prime }}_{j}^{2}E\left(\psi_{j}^{2}\left(\xi |\xi_{I}\right)\right).$$

Specifically, for main effect indices, since we have ***I*** = {*i*}, Equation (29) can be further rewritten as:

(32) $$g_{PCE}\left(\xi_{\sim [i]}\right)=\sum_{j=0}^{N^{\prime }-1}{c^{\prime }}_{j}\psi_{\alpha }\left(\xi_{\sim [i]}\right).$$

and for total effect indices, since we have ***I*** = {1, …, *n*}/{*i*}, Equation (29) can be further rewritten as:

(33) $$g_{PCE}\left(\xi_{i}\right)=\sum_{j=0}^{N^{\prime }-1}{c^{\prime }}_{j}\phi_{\alpha }\left(\xi_{i}\right).$$

The C-PCE could provide the first two order moments accurately. However, there are some difficulties for high order moment calculation. Consider the fact that the C-PCE consists of orthogonal bases, the products of two C-PCEs could also be expanded as a linear combination of orthogonal bases [29]. Therefore, the orthogonal characteristic of PCE multiplication remains. The generalization form of orthogonal bases are presented in [29].

Take the C-PCE with Hermite polynomial bases for example, suppose two C-PCEs $u=\sum_{\left|\alpha \right|\leq p_{\alpha }}{c^{\prime }}_{\alpha }\psi_{\alpha }\left(\xi |\xi_{I}\right)$ and $v=\sum_{\left|\beta \right|\leq p_{\beta }}{c^{\prime }}_{\beta }\psi_{\beta }\left(\xi |\xi_{I}\right)$. If $E\left({\left|uv\right|}^{2}\right)<\infty$, then the product of *u* and *v* has the PCE formula as:

(34) $$uv=\sum_{\left|\theta \right|\leq p_{\alpha }+p_{\beta }}\sum_{0\leq \beta \leq \theta }\sum_{\begin{array}{l} \left|\theta -\beta +r\right|\leq p_{\alpha },\\ \left|\beta +r\right|\leq p_{\beta } \end{array}}C\left(\theta ,\beta ,r\right)u_{\theta -\beta +r}v_{\beta +r}\psi_{\theta }\left(\xi \right),$$

where:

(35) $$C\left(\theta ,\beta ,r\right)={\left[\left(\begin{array}{c} \theta -\beta +r\\ r \end{array}\right)\left(\begin{array}{c} \beta +r\\ r \end{array}\right)\left(\begin{array}{c} \theta \\ \theta -\beta \end{array}\right)\right]}^{\frac{1}{2}}.$$

The subscripts ***α***, ***β***, ***r***, and ***θ*** in Equations (34) and (35) are tuples associated with C-PCE terms, e.g., ***α*** = (*α*_1_, *α*_2_, …, *α_n_*). We say ***β*** ≤ ***θ*** if *β_i_* ≤ *θ_i_* for all *i* = 1, 2, …, *n*. The operation of these subscripts, such as + or −, is also defined as component-wise. Especially, the factorial of tuples is defined like ***α***! = ∏*_i_ α_i_*!.

Then, the mean of *uv* could be obtained as:

(36) $$E\left(uv\right)=\sum_{\left|r\right|\leq \min(p_{\alpha },p_{\beta })}C\left(\theta =0,\beta =0,r\right)u_{r}v_{r}=\sum_{\left|r\right|\leq \min(p_{\alpha },p_{\beta })}u_{r}v_{r}.$$

Based upon the preparation above, we could calculate the high-order moments of PCE by replacing *u* and *v* with two PCEs respectively. That is, *u* and *v* in Equation (34) could be replaced by $g_{PCE}\left(\xi |\xi_{I}\right)$, $g_{PCE}^{2}\left(\xi |\xi_{I}\right)$, $g_{PCE}^{3}\left(\xi |\xi_{I}\right)$, …, respectively as Equation (37):

(37) $$\begin{array}{l} E\left(y^{2}\left(\xi |\xi_{I}\right)\right)\approx E\left(g_{PCE}^{2}\left(\xi |\xi_{I}\right)\right)=E\left(g_{PCE}\left(\xi |\xi_{I}\right)\cdot g_{PCE}\left(\xi |\xi_{I}\right)\right)\\ E\left(y^{3}\left(\xi |\xi_{I}\right)\right)\approx E\left(g_{PCE}^{3}\left(\xi |\xi_{I}\right)\right)=E\left(y_{p}^{2}\left(\xi |\xi_{I}\right)\cdot g_{PCE}\left(\xi |\xi_{I}\right)\right)\\ \;\vdots \\ E\left(y^{k}\left(\xi |\xi_{I}\right)\right)\approx E\left(g_{PCE}^{k}\left(\xi |\xi_{I}\right)\right)=E\left(g_{PCE}^{\left\lfloor \frac{k}{2}\right\rfloor }\left(\xi |\xi_{I}\right)\cdot g_{PCE}^{\left\lceil \frac{k}{2}\right\rceil }\left(\xi |\xi_{I}\right)\right), \end{array}$$

where ⌊•⌋ is rounded down and ⌈•⌉ is rounded up. Thus, the high order moments could be computed analytically.

### 3.3. The Maximum Entropy Method

Let *f_y_*(*y*) be the unknown PDF of the output of limit-state function and assume *H*(*f_y_*(*y*)) to be the entropy of *f_y_*(*y*). Then we have:

(38) $$H\left(f_{y}\left(y\right)\right)=-\int_{y\in \Omega_{y}}f_{y}\left(y\right)\ln\left(f_{y}\left(y\right)\right)dy,$$

where *Ω_y_* is the domain of *y*. According to the principle of ME, the failure probability distribution which best represents the current information, such as the moments, is the one with the largest entropy [24]. Thus, the ME method for estimating *f_y_*(*y*) can be formulated as:

(39) $$\begin{array}{l} \max_{f_{y}\left(\cdot \right)}H\left(f_{y}\left(y\right)\right)\\ s.t.\;\int_{y\in \Omega_{y}}f_{y}\left(y\right)dy=1\\ \;\int_{y\in \Omega_{y}}y^{k}f_{y}\left(y\right)dy=\mu_{k},\;k=1,2,\cdots ,N_{ME}, \end{array}$$

where *N_ME_* is the number of moments, and *μ_k_* is the *k*th moment, i.e., *μ*_1_ = *E*(*y*), *μ*_2_ = *E*(*y^2^*), etc.

It can be proved that the optimal solution for the PDF formula is given as:

(40) $$f_{y}\left(y\right)=\exp\left(-\sum_{i=0}^{N_{ME}}\lambda_{i}y^{i}\right).$$

Thus, *λ_i_* in Equation (40) should satisfy the following equation:

(41) $$\begin{array}{l} \int_{y\in \Omega_{y}}e^{-\sum_{i=0}^{N}\lambda_{i}}dy=1\\ \int_{y\in \Omega_{y}}y^{k}e^{-\sum_{i=0}^{N}\lambda_{i}}dy=\mu_{k};\;k=1,2,\cdots ,N. \end{array}$$

Specifically, if the first two moments of the output are available, we have:

(42) $$\lambda_{0}^{*}=\frac{E{\left(y\right)}^{2}}{2V\left(y\right)}+\frac{1}{2}\ln\left(2\pi V\left(y\right)\right),\;\lambda_{1}^{*}=-\frac{E\left(y\right)}{V\left(y\right)},\;\lambda_{2}^{*}=\frac{1}{2V\left(y\right)},$$

where $V\left(y\right)=E\left(y^{2}\right)-E{\left(y\right)}^{2}$ and the failure probability can be calculated as:

(43) $$P_{f}=\frac{1}{2}\frac{\sqrt{\pi }\exp\left(\frac{1}{4}\frac{{\left(\lambda_{1}^{*}\right)}^{2}}{\lambda_{2}^{*}}\right)\left(erf\left(\frac{1}{2}\frac{\lambda_{1}^{*}}{\sqrt{\lambda_{2}^{*}}}\right)+1\right)\exp\left(-\lambda_{0}^{*}\right)}{\sqrt{\lambda_{2}^{*}}}.$$

In the proposed method, if taking Equations (27) and (28) into Equations (42) and (43), the failure probability of C-PCE could be obtained directly without additional sampling.

For more general cases, the first four moments are usually used for PDF calculation because they are corresponding to mean, variance, skewness, kurtosis respectively, which are clear in physics meaning [30]. The 3rd moment and the 4th moment could be obtained efficiently by Equation (37) instead of traditional sampling method. Besides, since the ME method may not have the closed form solution, some optimization techniques, such as Newton method or its improved versions [31,32], could be utilized to solve the failure probability.

### 3.4. Global Reliability Sensitivity Indices Calculation

Let ${\left\{\xi_{I}^{\left(i\right)}\right\}}_{i=1}^{M_{R}}$ denote a set of conditional variable samples, where *M_R_* is the number of samples. As described above, when the PCE for limit-state function is given, if taking each conditional variable sample into Equation (24), it returns the mean and variance of C-PCE. Then, the CFP is calculated by ME method. In this way, we could get a set of surrogate model relationship between ${\left\{{\left[\begin{array}{ccc} \psi_{0}\left(\xi_{I}^{\left(i\right)}\right) & \cdots & \psi_{N}\left(\xi_{I}^{\left(i\right)}\right) \end{array}\right]}^{T}\right\}}_{i=1}^{M_{R}}$ and ${\left\{P_{f}\left(\xi_{I}^{\left(i\right)}\right)\right\}}_{i=1}^{M_{R}}$. Similar to the traditional PCE procedure as Equation (A7) in Appendix B, we could solve the coefficients of R-PCE. Then, the result of R-PCE is:

(44) $$P_{f|X_{I}}=E\left[I_{F}|x_{I}\right]=P_{f}\left(x_{I}\right)=P_{f}\left(\xi_{I}\right)=\sum_{j=0}^{N_{R}-1}c_{R,j}\psi_{j}\left(\xi_{I}\right),$$

where *c*_*R*,*j*_ is *j*th coefficient of R-PCE, and the subscript “*R*” means the coefficient is used for GRSA. ***ξ_I_*** is independent standardized random variables corresponding to original variables ***x_I_***, *N_R_* is the total number of coefficients, which is determined by:

(45) $$N_{R}=\frac{\left(\left|I\right|+p_{R}\right)!}{\left|I\right|!p_{R}!},$$

where *p_R_* is the predefined degree of R-PCE. Then, the CFP is:

(46) $$P_{f}=E\left(P_{f}\left(\xi_{I}\right)\right)=c_{R,0}.$$

And the variance of CFP is:

(47) $$V\left(P_{f}\left(\xi_{I}\right)\right)=\sum_{j=1}^{N_{R}-1}c_{R,j}^{2}E\left(\psi_{j}^{2}\left(\xi_{I}\right)\right).$$

Substituting Equations (46) and (47) into Equations (22) and (23), we could calculate the main effect and the total effect of global reliability sensitivity indices.

### 3.5. Implementation

For a given limit-state function *g* = *g*(***x***), we assume here that the input random variables are independent. With preparation mentioned above, the complete algorithm description is introduced as follows:

*Step 1*: Represent distributions of the input into the associated standardized random variable. If the distribution types are not unified, we could map different distributions into the normal distribution. The following computation will be based upon standardized random variables.
*Step 2*: Compute PCE for limit-state function by Equation (A7) in Appendix B, and it refers as *g_PCE_*(***ξ***).
*Step 3*: Based upon PCE above, compute R-PCE. First of all, generate a set of conditional variable samples. Then, integrate *g_PCE_*(***ξ***) and ME method to obtain the CFP value *P_f_*(***ξ***|***ξ_I_***), where ***I*** = {*i*} for main effect indices and ***I*** = {1, 2, ..., *n*}/{*i*} for total effect indices. Specifically, for each sample set $\left\{\xi_{I}^{\left(j\right)}\right\}$, substitute it into Equations (24)–(26) to obtain C-PCE $g_{PCE}^{\left(j\right)}\left(\xi |\xi_{I}\right)$. With the moments given by C-PCE, we could calculate the $P_{f}\left(\xi_{I}^{\left(j\right)}\right)$ with ME method analytically. Third, the R-PCE comes out as Equation (44).
*Step 4*: Compute the global reliability sensitivity indices by Equation (22), Equation (23) and Equations (46) and (47).

The step-by-step procedure of algorithm for the main effect indices and the total effect indices by the proposed 2-layer PCE method are presented in Algorithms A1 and A2 in Appendix C respectively. The total evaluations of the limit-state function is $\frac{2\left(n+p\right)!}{n!p!}+1$, where *n* and *p* are input variable dimension and the PCE degree respectively. If taking times of PCE evaluation in R-PCE construction process into consideration, the computation costs are $\frac{2\left(n+p\right)!}{n!p!}+2np_{R}+\frac{2\left(n+p_{R}-1\right)!n}{\left(n-1\right)!p_{R}!}+3+n$, where *p_R_* is the degree of R-PCE. This computation burden is usually 1–2 times less than IS procedure and 2–3 times less than MCS procedure, which could be seen in the following test examples. And the efficiency of the proposed method could be further improved by adopting sparse algorithms [6,17,33]. However, such work is not the scope of this paper.

## 4. Test Examples

Implementation of the proposed method for global reliability sensitivity indices calculation is illustrated by two test examples. The first example is a numerical example, while the second one is a structural analysis of a bridge crane. The results obtained by the proposed method are compared with solutions obtained by the full-scale MCS and the IS method from the perspective of accuracy and efficiency respectively.

### 4.1. A Numerical Example

Consider a limit-state function:

(48) $$g\left(x\right)=10-18x_{1}+x_{2}^{2}+x_{2}+x_{3}^{2}+5x_{3},$$

where the input variables follow the normal distribution or the lognormal distribution, i.e., ln*x*_1_~N (0.5,0.1), ln*x*_2_~N (0.6,0.1), and ln*x*_3_~N (0.5,0.1). The proposed method is employed to compute the main effect indices $S_{i}^{\left(R\right)}$ and total effect indices $S_{T_{i}}^{\left(R\right)}$, when the PCE degree for the limit-state function is 2, and the R-PCE degree for CFP is 7. The results are compared with the results obtained by the full-scale MCS method and the IS method in Table 1. Moreover, the total times of limit-state function evaluation by different methods are also shown in the last column. It could be seen that the relative error of both methods are less than 3%, and about half of the results calculated by the proposed method is better than those obtained by the IS method. Moreover, the computational cost of proposed method is reduced sufficiently.

**Figure 2:**
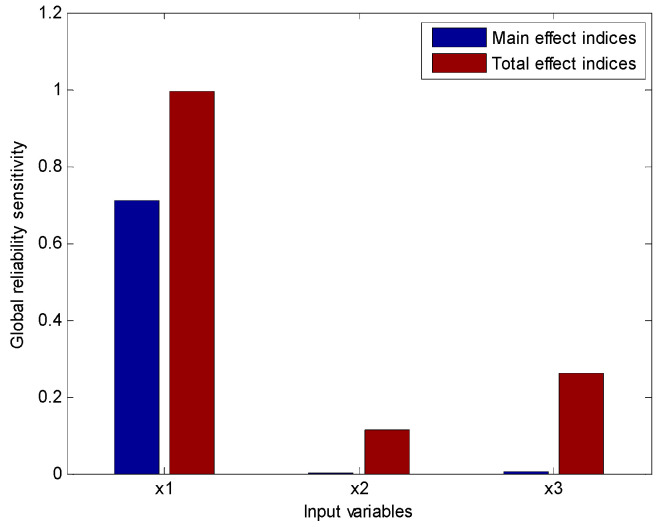
The importance ranking based on global reliability sensitivity indices.

Also, the main effect indices and the total effect indices of GRSA are schematically shown in Figure 2. We could see that both the main effect indices $S_{i}^{\left(R\right)}$ and the total effect indices $S_{T_{i}}^{\left(R\right)}$ produce the same importance ranking: *x*_1_ > *x*_3_ > *x*_2_, and there are strong interaction effects among these input variables since the total effects are much larger than the main effects. This suggests that the risk may be reduced by decreasing the uncertainty of *x*_1_.

### 4.2. An Engineering Example

**Figure 3:**
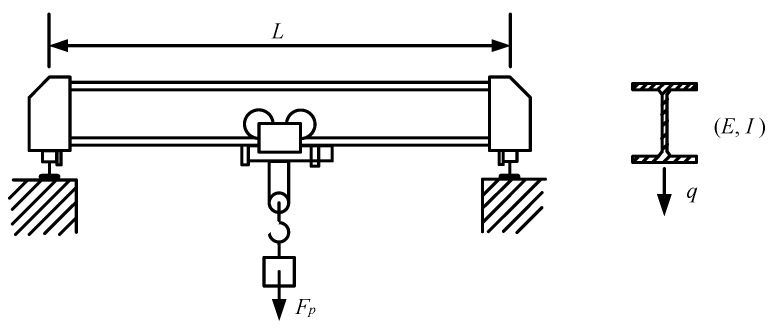
The beam structure of example 2.

The second example is a bridge crane with respect to its rigidity design in Figure 3. The crane is made by universal beam with movable sheaves and hook. When the weight is in the middle of the beam, the maximal deflection $\omega_{\max}$ of such a crane beam could be computed as:

(49) $$\omega_{\max}\left(q,E,I,L,F_{p}\right)=\frac{L^{3}}{EI}\left(\frac{F_{p}}{48}+\frac{5qL}{384}\right),$$

where *q* is the magnitude of a normally distributed load that represents the dead-weight of crane beam, *E* and *I* denote Young’s modulus and inertia moment of universal beam respectively, *L* is the span length, and *F_p_* is maximal weight including listed load, sheaves and hook. Two distribution types are taken for this example, namely, the normal distribution and the lognormal distribution. Besides, two different variances are also considered to test this example, which are labeled as case1 and case 2 respectively. The detail distribution parameters are given in Table 2.

According to design requirement, the admissible ratio of deflection to span length is lower than 0.0018. Thus, the limit-state function is given as:

(50) $$g\left(q,E,I,L,F_{p}\right)=0.0018-\frac{\omega_{\max}}{L}=0.0018-\frac{L^{2}}{EI}\left(\frac{F_{p}}{48}+\frac{5qL}{384}\right).$$

The results obtained by the full-scale MCS method, the IS method and the proposed method are given in Table 3 and Table 4. The full-scale MSC results with large sample size are seen as the accurate results. In the calculation procedure of the proposed method, the PCE degree for the limit-state function and the PCE degree for CFP are 5 and 7 respectively.

It is shown that both the IS method and the proposed method could approximate $S_{i}^{\left(R\right)}$ and $S_{T_{i}}^{\left(R\right)}$ with much less computational burden, and the latter one presents better efficiency than the former one. In case 1, the average relative error of global reliability sensitivity indices obtained by proposed method is about 0.0196, while that obtained by the IS method is about 0.0283. In case 2, the average relative error of global reliability sensitivity indices obtained by proposed method is about 0.0206, while that obtained by the IS method is about 0.0295. The computational costs of our method are about two orders of magnitude less than the IS method, and four orders of magnitude less than the MCS method. Thus, the proposed method may be better than the other two methods.

**Figure 4:**
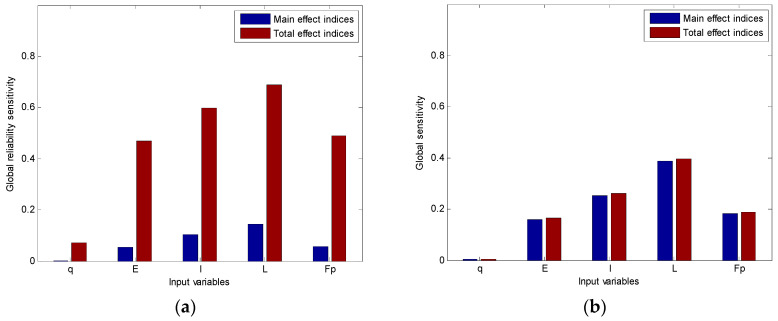
The importance ranking of case 1. (**a**) The importance ranking based on GRSA; (**b**) The importance ranking based on GSA.

**Figure 5:**
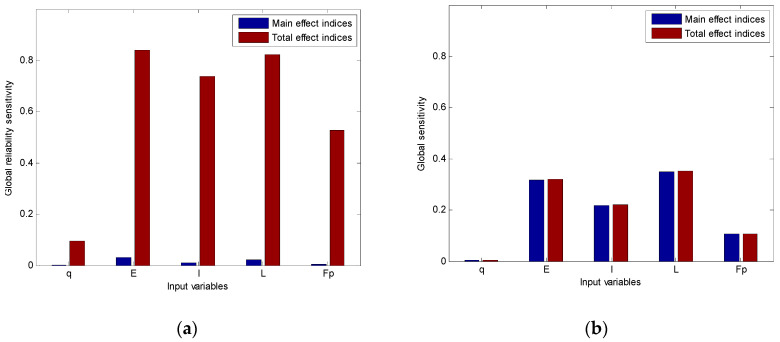
The importance ranking of case 2. (**a**) The importance ranking based on GRSA; (**b**) The importance ranking based on GSA.

Moreover, the global reliability sensitivity indices are also schematically shown in Figure 4a and Figure 5a. In case 1, the importance ranking based upon global reliability sensitivity indices $S_{i}^{\left(R\right)}$ and $S_{T_{i}}^{\left(R\right)}$ are given as *L* > *I* > *F_p_* > *E > q*. It indicates that the reliability could be increase mostly by decreasing the uncertainty of the span length. Besides, since the total effect indices of input variables are much higher than their main effect indices, there are strong interaction effects among these variables. In case 2, the importance ranking based upon global reliability sensitivity indices $S_{i}^{\left(R\right)}$ and $S_{T_{i}}^{\left(R\right)}$ is given as *E* > *L > I* > *F_p_ > q*. Obviously, the influence of Young’s modulus on failure probability is higher than other random variables, which is different from case 1. Moreover, strong interaction effects also exist in this case. However, the interaction effects make more contributions than those in case 1 even when input variances are smaller compared with those of case 1. Therefore, the importance ranking should be paid more attention during further optimization design.

The global sensitivity indices of maximal deflection of cases 1 and 2 are also computed in Table 5 by traditional GSA method mentioned in Section 2.1, and they are plotted in Figure 4b and Figure 5b respectively. It is shown that the results of global sensitivity indices and global reliability sensitivity indices may be different. First of all, although the importance ranking based on GSA of case 1 is the same as that based on GRSA, the importance ranking obtained by GSA of case 2 is *L* > *E* > *I* > *Fp* > *q*, which is different from that obtained by GRSA. Secondly, the total effect indices of both cases are similar to the main effect indices, which means that there are few interaction effects among input variables. This is also different from the results of GRSA.

Comparing the results of GRSA with those of GSA, it suggests that the importance of input variables on the failure probability is not equivalent to that on the model output. So, both global reliability sensitivity indices and global sensitivity indices may be considered in engineering practice when choosing importance variables. And the proposed method could provide a useful tool for such purpose.

## 5. Conclusions

Global reliability sensitivity analysis aims at measuring the importance statistical parameter with regards to the input variable. The variance of expectation of conditional indicator function in global reliability sensitivity indices is difficult to calculate. This paper develops a PCE-based GRSA method to solve the problem. The proposed method takes conditional failure probability to replace the expectation of conditional indicator function, and then utilizes a 2-layer PCE construction technology to obtain the variance of conditional failure probability. The efficiency of proposed method is illustrated by a numerical example and an engineering model example. Both main effect indices and total effect indices present good performance in accuracy and significantly less computational burden in limit-state function evaluations compared with the traditional full-scale MCS method and IS method. Through global reliability sensitivity analysis, we could determine which uncertainties of inputs variables should be controlled and pave the way for further design improvement or optimization.

## Figures and Tables

**Table 1:** The global reliability sensitivity indices of example 1.

Method		*x*_1_	*x*_2_	*x*_3_	Times of Function Evaluations
**Full-scale MCS**	$S_{i}^{\left(R\right)}$	0.7120	0.0005274	0.005265	1.5 × 10^7^
	$S_{T_{i}}^{\left(R\right)}$	0.9946	0.1130	0.2602	
**IS**	$S_{i}^{\left(R\right)}$	0.7206	0.0005147	0.005327	1.2 × 10^5^
	$S_{T_{i}}^{\left(R\right)}$	0.9945	0.1124	0.2583	
**Proposed method**	$S_{i}^{\left(R\right)}$	0.7181	0.0005426	0.005139	182
	$S_{T_{i}}^{\left(R\right)}$	0.9932	0.1119	0.2625	

**Table 2:** The distribution parameters of the input variables of example 2.

Input Variables		*q*/(*N*/*M*)	*E*/(*kN*/*m*^2^)	*I*/*m*^4^	*L*/m	*F_p_*
**Distribution type**		Normal	Normal	Normal	Lognormal	Lognormal
**Mean value**		662.5	2 × 10^11^	2.172 × 10^−4^	10	3 × 10^4^
**variances**	**Case 1**	0.1	0.08	0.1	0.06	0.1
	**Case 2**	0.05	0.06	0.05	0.03	0.04

**Table 3:** The global reliability sensitivity indices of case 1.

Method		*q*	*E*	*I*	*L*	*F_p_*	Times of Function Evaluations
**Full-scale MCS**	$S_{i}^{\left(R\right)}$	0.001283	0.05398	0.1029	0.1446	0.05583	1.5 × 10^7^
	$S_{T_{i}}^{\left(R\right)}$	0.07086	0.4701	0.5967	0.6891	0.4895	
**IS**	$S_{i}^{\left(R\right)}$	0.001267	0.05103	0.09755	0.1397	0.05129	1.2 × 10^5^
	$S_{T_{i}}^{\left(R\right)}$	0.06961	0.4727	0.6026	0.6985	0.4905	
**Proposed method**	$S_{i}^{\left(R\right)}$	0.001118	0.05404	0.1031	0.1445	0.05591	3882
	$S_{T_{i}}^{\left(R\right)}$	0.07251	0.4714	0.5879	0.6865	0.4983	

**Table 4:** The global reliability sensitivity indices of case 2.

Method		*q*	*E*	*I*	*L*	*F_p_*	Times of Function Evaluations
**Full-scale MCS**	$S_{i}^{\left(R\right)}$	0.000486	0.03123	0.01150	0.02078	0.002947	1.5 × 10^7^
	$S_{T_{i}}^{\left(R\right)}$	0.09433	0.8405	0.7389	0.8240	0.5270	
**IS**	$S_{i}^{\left(R\right)}$	0.000502	0.03111	0.01194	0.02260	0.002806	1.2 × 10^5^
	$S_{T_{i}}^{\left(R\right)}$	0.09845	0.8509	0.7356	0.8333	0.5336	
**Proposed method**	$S_{i}^{\left(R\right)}$	0.000532	0.03064	0.01183	0.02047	0.003107	3882
	$S_{T_{i}}^{\left(R\right)}$	0.09512	0.8497	0.7298	0.8199	0.5277	

**Table 5:** The global sensitivity indices of example 2.

		*q*	*E*	*I*	*L*	*F_p_*
Case 1	$S_{i}$	0.003538	0.15818	0.253083	0.387629	0.183083
	$S_{T_{i}}$	0.003718	0.16375	0.260962	0.397182	0.188962
Case 2	$S_{i}$	0.003182	0.317395	0.218915	0.349925	0.106503
	$S_{T_{i}}$	0.003227	0.319898	0.220892	0.352517	0.107553

## Appendix A

**Table A1:** Notation.

n	the dimension of a variable
x	$x=\left(x_{1},x_{2},\cdots ,x_{n}\right)$, the input of given model
ξ	$\xi =\left(\xi_{1},\xi_{2},\cdots ,\xi_{n}\right)$, the independent standardized orthogonal random variable
y	the output of given model
$x_{\sim \left[i\right]}\left(\xi_{\sim \left[i\right]}\right)$	the vector without the *i*th element
$f_{x}\left(x_{i}\right)$	the PDF of *x_i_*
$f_{x}\left(x\right)$	$f_{x}\left(x\right)=\prod_{i=1}^{n}f_{x}\left(x_{i}\right)$, the joint PDF of $x=\left(x_{1},x_{2},\cdots ,x_{n}\right)$
$f_{y}\left(y\right)$	the PDF of *y*
E(·)	expectation
V(·)	variance
$S_{T_{i}}$	the main effect of GSA
$S_{i}$	the total effect of GSA
$S_{i}^{\left(R\right)}$	the main effect of GRSA
$S_{T_{i}}^{\left(R\right)}$	the total effect of GRSA
p	the degree of PCE
$p_{R}$	the degree of R-PCE
$c_{j}$	the coefficient of *j*th PCE item
${c^{\prime }}_{j}$	the coefficient of *j*th CPCE item
$c_{R,j}$	the coefficient of *j*th R-PCE item
N	the number of PCE items
$N^{\prime }$	the number of CPCE items
$N_{R}$	the number of R-PCE items
$\phi_{k}$	the *k*th one-dimensional orthogonal polynomial
$\psi_{\alpha }\left(\xi \right)$	$\psi_{\alpha }\left(\xi \right)=\prod_{k=1}^{n}\phi_{\alpha_{k}}\left(\xi_{k}\right)$ where $\sum_{k=1}^{j}\alpha_{k}\leq p$ and $\alpha =\left(\alpha_{1},\alpha_{2},\cdots ,\alpha_{n}\right)$, the base of PCE
$I_{F}$	the indicator function
$P_{f}$	the failure probability
$\lambda_{i}$	the unknown parameter of ME formula
$N_{ME}$	the number of unknown parameter of ME formula
$\mu_{k}$	the *k*th moment of y

## Appendix B

Consider the model *y* = *g*(***x***) in Section 2.1 again. Then, let ${\left\{\xi_{i}\right\}}_{i=0}^{\infty }$ be independent standardized orthogonal random variables associated with ${\left\{x_{i}\right\}}_{i=1}^{n}$. For example, if *x_i_*~N (2,0.5), then we have *x_i_* = 0.5*ξ_i_* + 2 where *ξ_i_* is subject to the standardized normal distribution N (0,1).

Then, the PCE of the limit-state function is:

(A1) $$y=g_{PCE}\left(\xi \right)=c_{0}\psi_{0}+\sum_{i_{1}=1}^{n}c_{i_{1}}\psi_{1}\left(\xi_{i_{1}}\right)+\sum_{i_{1}=1}^{n}\sum_{i_{2}=1}^{i_{1}}c_{i_{1}i_{2}}\psi_{2}\left(\xi_{i_{1}},\xi_{i_{2}}\right)+\sum_{i_{1}=1}^{n}\sum_{i_{2}=1}^{i_{1}}\sum_{i_{3}=1}^{i_{2}}c_{i_{1}i_{2}i_{3}}\psi_{3}\left(\xi_{i_{1}},\xi_{i_{2}},\xi_{i_{3}}\right)+\cdots ,$$

where ${\left\{c_{j}\right\}}_{j=0}^{\infty }$ is the coefficient, and expansion base $\psi_{s}\left(\xi_{i_{1}},\cdots ,\xi_{i_{s}}\right)$ denotes the *s*th multi-dimensional hyper-geometric polynomials in terms of the multi-dimensional standardized random variable $\xi ={\left(\xi_{1},\cdots ,\xi_{n}\right)}^{T}$. It is defined as tensor products of the corresponding one-dimensional orthogonal polynomials ${\left\{\phi_{k}\right\}}_{k=0}^{\infty }$:

(A2) $$\psi_{s}\left(\xi_{1},\xi_{2},\cdots ,\xi_{s}\right)=\prod_{k=1}^{s}\phi_{\alpha_{k}}\left(\xi_{k}\right),$$

where *ϕ_i_* is an one-dimensional orthogonal basis with orthogonality relation

(A3) $$\langle \phi_{i},\phi_{j}\rangle =\langle \phi_{i}^{2}\rangle \delta_{ij},$$

where *δ_ij_* is the Kronecker delta and 〈⋅,⋅〉 denotes the ensemble average which is the inner product in Hilbert space. And *α_k_* is the vector of index, which is subjected to a nonnegative integer.

The type of polynomials depends on the distributed type of input variables, e.g., the orthogonal polynomials corresponding to normal distribution are Hermite polynomials. For more details about interpretation of polynomial terms see [32]. It should be noted that, if the distribution types of input variables are not the same, Isukapalli [26] provided several distribution transformation approaches that map different distributions as functions of normal random variables. With this pre-processing, the problem of inconsistent distribution could be overcome.

In engineering practice, the PCE is truncated to finite terms. So, considering an *n*-dimensional orthogonal polynomial with degree not exceeding *p*, Equation (A1) can be rewritten as another form with limited input uncertainties, that is,

(A4) $$\begin{array}{ll} y\approx g_{PCE}\left(\xi \right)= & c_{0}+\sum_{i=1}^{n}\sum_{\alpha \in \varphi_{i}}c_{\alpha }\psi_{\alpha }\left(\xi_{i}\right)+\sum_{1\leq i_{1}<i_{2}\leq n}\sum_{\alpha \in \varphi_{i_{1}i_{2}}}c_{\alpha }\psi_{\alpha }\left(\xi_{i_{1}},\xi_{i_{2}}\right)+\cdots \\ & +\sum_{1\leq i_{1}<\cdots <i_{s}\leq n}\sum_{\alpha \in \varphi_{i_{1}\cdots i_{s}}}c_{\alpha }\psi_{\alpha }\left(\xi_{i_{1}},\cdots ,\xi_{i_{s}}\right)+\cdots +\sum_{\alpha \in \varphi_{1,2,\cdots ,n}}c_{\alpha }\psi_{\alpha }\left(\xi_{1},\cdots ,\xi_{n}\right), \end{array}$$

where the subscript ***α*** is a tuple defined as ***α*** *=* (*α_1_*, …, *α_n_*), and $\varphi_{i_{1},\cdots ,i_{s}}$ is defined as a realization of ***α*** tuple so that only the indices {*i*_1_, …, *i_s_*} are nonzero:

(A5) $$\varphi_{i_{1},\cdots ,i_{s}}=\left\{\alpha :\begin{array}{c} \alpha_{k}>0\;\forall k=1,2,\cdots ,n.\;k\in \left(i_{1},\cdots ,i_{s}\right)\\ \alpha_{k}=0\;\forall k=1,2,\cdots ,n.\;k\notin \left(i_{1},\cdots ,i_{s}\right) \end{array}\right\}.$$

Correspondingly, the expansion base $\psi_{\alpha }\left(\xi \right)=\prod_{k=1}^{n}\phi_{\alpha_{k}}\left(\xi_{k}\right)$, where $\sum_{k=1}^{j}\alpha_{k}\leq p$. Denote *N* as the total number of polynomials, then we have $N=\left(\begin{array}{c} n+p\\ p \end{array}\right)=\frac{\left(n+p\right)!}{n!p!}.$

Generally, Equation (A4) can be further simplified as

(A6) $$y\approx g_{PCE}\left(\xi \right)=\sum_{j=0}^{N-1}c_{j}\psi_{j}\left(\xi \right),\;for\;\xi =\left(\xi_{1},\cdots ,\xi_{n}\right),$$

where coefficient *c_j_* and expansion base *ψ_j_*(***ξ***) are corresponding to Equation (A4) sequentially.

Let ${\left\{\xi^{i}\right\}}_{i=1}^{M}$ denote a set of the random variable samples, and it could be determined by the probabilistic collocation method (PCM). Again Let ${\left\{g\left(\xi^{i}\right)\right\}}_{i=1}^{M}$ denote the corresponding set of model output, where *M* is the number of samples. Denote $c={\left(c_{0},\cdots ,c_{N-1}\right)}^{T}$, thus an approximation $\hat{c}$ is given by:

(A7) $$\hat{c}=\arg\underset{c}{\min}\sum_{i=1}^{M}{\left(g\left(\xi^{i}\right)-\sum_{j=0}^{N}c_{j}\psi_{j}\left(\xi^{i}\right)\right)}^{2}={\left(\psi^{T}\psi \right)}^{-1}\psi^{T}y\left(\xi^{1:M}\right),$$

where $\psi ={\left\{{\left[\begin{array}{ccc} \psi_{0}\left(\xi^{\left(i\right)}\right) & \cdots & \psi_{N}\left(\xi^{\left(i\right)}\right) \end{array}\right]}^{T}\right\}}_{i=1}^{M}$ denotes the coefficient matrix and $y\left(\xi^{1:M}\right)={\left(g\left(\xi^{1}\right),\cdots ,g\left(\xi^{M}\right)\right)}^{T}$. Since $\psi^{T}\psi$ may be ill-conditioned, *M* is suggested to be selected as *M* = 2(*N* + 1) [18].

Due to the orthogonality of the basis, the mean value and the variance of *y* in Equation (A6) can be calculated as:

(A8) $$\begin{array}{l} E\left(y\right)=c_{0},\\ V\left(y\right)=\sum_{j=1}^{N-1}c_{j}^{2}E\left(\psi_{j}^{2}\left(\xi \right)\right). \end{array}$$

It could be proved that the PCE-based main effect index *S_i_* and total effect index $S_{T_{i}}$ are derived as [6]:

(A9) $$S_{i}=\frac{\sum_{\alpha \in \varphi_{i}}c_{\alpha }^{2}E\left[\psi_{\alpha }^{2}\right]}{V\left(y\left(\xi \right)\right)}$$

and:

(A10) $$S_{T_{i}}=\sum_{\left(i_{1},\cdots ,i,\cdots ,i_{s}\right)\in \varphi_{i_{1},\cdots ,i_{s}}}S_{i_{1},\cdots ,i,\cdots i_{s}}.$$

Therefore, the PCE could obtain the expectation and variance of the model output efficiently. Moreover, compared with the traditional MCS method which requires a large number of samples [16], the PCE method could calculate Sobol’s indices with quit a few computation costs. In this way, PCE is widely employed for GSA application.

## Appendix C

**Algorithm A1:** Algorithm for main effect indices using proposed method.

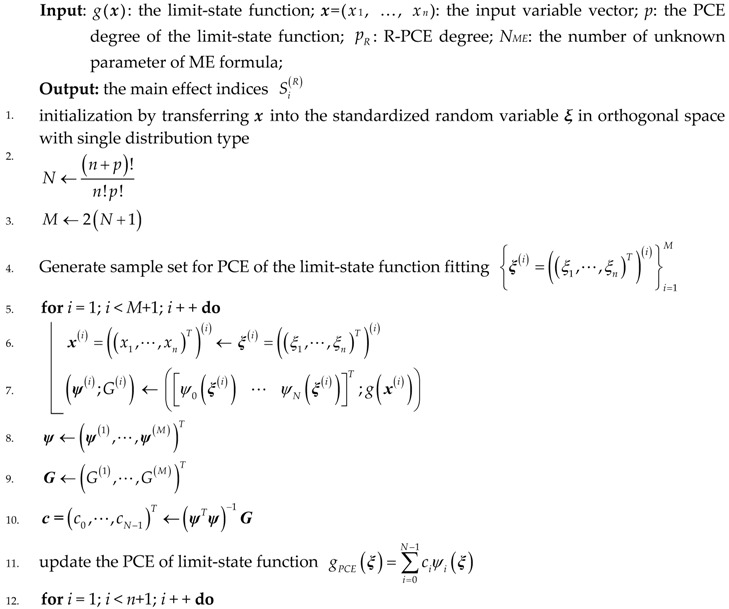
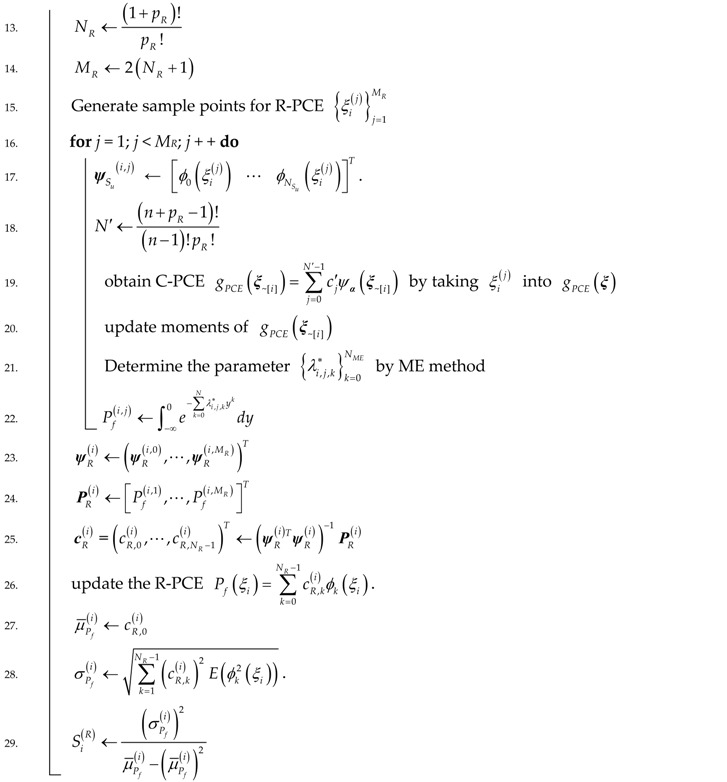

**Algorithm A2:** Algorithm for Total Effect Indices Using Proposed Method.
